# Nurses' Evaluation of Their Use and Mastery in Health Assessment Skills: Selected Iran's Hospitals

**DOI:** 10.5812/nms.13316

**Published:** 2013-09-15

**Authors:** Mohsen Adib-Hajbaghery, Azade Safa

**Affiliations:** 1 Trauma Nursing Research Center, Kashan University of Medical Sciences, Kashan, IR Iran; 2 Medical Surgical Nursing Department, Faculty of Nursing and Midwifery, Kashan University of Medical Sciences, Kashan, IR Iran

**Keywords:** Health assessment, Clinical education, Nursing, Clinical skills

## Abstract

**Background:**

Health assessment skills are of the most important skills which nurses require. The more precise assessment, the better results would be obtained and the quality of patient care would be improved. However, in Iran, few studies have investigated nurses’ assessment skills.

**Objectives:**

This study was aimed to assessnurses' evaluation of the learned skills of health assessment and their use.

**Materials and Methods:**

This cross-sectional study was conducted on 200 nurses in Isfahan province hospitals. Data was collected by a questionnaire including demographic data and 120 health assessment skills. Nurses scored their frequency of using and proficiency in skills. Statistical analysis was conducted by ANOVA, Tukey test and independent sample T-tests.

**Results:**

The highest level of using and proficiency in skills was related to taking history. Nurses received 87.25% of score in this field. The lowest level of application was in assessment of the urogenital system so that nurses received 16.37% of score in this area. Also the lowest proficiency was in assessment of the nervous system and nurses received 34.58% of score in this area.

**Conclusions:**

The level of nurses' proficiency in the health assessment skills was not satisfactory. Modifying the curriculum and cooperating of nurse managers and nursing schools can help to improve the situation.

## 1. Background

Health assessment is a key element in nursing process (1, 2). These skills play a decisive role in assessing and determining the patients’ health problems and caring needs and consequently have a crucial role in designing nursing care plans and determining the nursing interventions. The higher levels of health assessment skills would increase nurses’ capability to monitor the changes in patients’ health and contributes to make better judgments and nursing diagnosis (3, 4). The more precise assessment, the better results would be obtained and the quality of patient care would be improved (5). Thus, ensuring the nurses and nurse students in these skills is an important issue (3). 

Assessment of clinical skills is usually performed by nursing instructors (6). However, nurses and nurse students self-evaluation of their caring skills is stressed in recent years. It is believed that monitoring the professionals’ behavior with self-assessment checklists is not only reliable but also may help them develop meta-cognitive skills, assist them becoming more independent and confident and empower them to select higher goals and to try to realize these goals and finally assist them to improve and strengthen their skills (7, 8).

Nonetheless, some studies have shown that nurses neither have enough skills to evaluate patients nor can use these skills correctly. In a study, conducted on American nurses, from a total of 120 skills of health assessment, 29% has been used daily or weekly, 34% has been used monthly or occasionally and 37% has never been used by nurses (9). In another study conducted on 1220 Austrian nurses, from 120 health assessment skills 34% has been used routinely, 35% has been used occasionally and 31% has been used rarely by nurses (10). Also, a recent study conducted on nursing students has reported that from a total of 124 physical examination skills, only 30 skills were used regularly while the other ones have been used rarely or never (11).

In Iran, few studies have investigated nurses’ assessment skills. Bahreiny et al. assessed the clinical competence of nurses worked in Shiraz hospitals and reported that nurses have good health assessment skills (12). However Madani et al. investigated the senior nursing students' health assessment skills in Zanjan University and reported that most of the senior nursing students were deficient in health assessment skills and only 11.4% of them had good skills (12).

## 2. Objectives

Regarding the lack of study on nurses’ health assessment skills and due to the key role of health assessment skills in providing necessary data to formulate caring plans, and also conflicting reports in this regard, the present study was conducted to evaluate nurses’ opinions on their own use and mastery in health assessment skills.

## 3. Materials and methods

This study was conducted in fall of 2012 using a cross-sectional method. Sampling was performed randomly among nurses worked in five selected hospitals in Isfahan province, Iran. Sample size was calculated based on the Cochran's formula with the type I error of 0.05, sampling error of 0.07, and the estimated prevalence of 0.6 (13). Then 188 samples were estimated to be needed; however, 200 people were selected to compensate for possible attrition. After calculating the sample size, a list of teaching hospitals of the province was provided and seven hospitals were selected randomly. Then the second author referred to the authorities to explain the study purposes and obtaining their permissions. The authorities of five hospitals gave permissions and provided the researcher a list of nurses in their hospital that was used as the sampling framework. Then, needed sample from each hospital was calculated based on the number of nursing staff in each and the needed samples in every hospital were selected randomly using a random number table. If a selected nurse did not accept to take part, a new sample was replaced randomly.

The inclusion criteria were having a bachelor or a master of science in nursing, employing in nursing position, and consent to enter the study. The questionnaire was given the participants and they wanted to fill it out and return it after one day. The questionnaire was designed through literature review and its content validity was confirmed by 10 nursing instructors. Reliability of the questionnaire was set at 0.83 by Cronbach’s Alpha.

The first part of the questionnaire included questions regarding personal information (i.e. age, sex, education level, professional working experience, working unit, and place of education). The second part included 120 health assessment skills which were evaluated in two columns. In the first column, skills were evaluated based on the frequency of using (as never, once in a few months, monthly, weekly, and daily with a score from 0 to 4 respectively). In the second column skills were evaluated based on individual proficiency in doing them that scored in a 4 choice Likert scale from ‘I am not proficient’ = 0, to ‘I am completely proficient’ = 3, respectively. Questions were in 15 domains including taking the health history (6 items), and assessment of bodily systems including the skin (4 items), head and neck (14 items), ear, nose and eyes (27 items), respiratory system (8 items) cardiovascular system (17 items), digestive system (12 items), musculoskeletal system (10 items), nervous system (12 items), and urogenital system (10 items). 

The range of skill score in the first column was zero to 480 and in the second column it was from zero to 360. Nurses who got 100% of the skill score (score = 480) were considered fully skilled. Also, a score higher than 50% of the total score was considered as desired and a score lower than 50% was considered as undesirable.

### 3.1. Data Analysis

Statistical analysis was conducted using SPSS software, version 11.5. Analysis of variance with Tukey test, independent sample T tests and Pearson correlation coefficient were used to analyze the data. The significance level was set at (P < 0.05) in all testes.

### 3.2. Ethical Considerations

This study was approved in the research council of the faculty of nursing and midwifery and its ethical aspects were approved by the research ethics committee in the Kashan University of Medical Sciences. Also, necessary licenses were obtained from the authorities of the concerned hospitals. The purposes of the study were explained to all of the participants and they signed the written informed consent and assured of the confidentiality of their personal information.

## 4. Results

In total, 146 (73%) of the participants were female, 68 (34%) had less than five years working experience and the others had more than five years of professional experience. The mean age of the participants was 31.46 ± 5.63 years. In total, 57 participants (28.5%) were working in medical units, 50 (25%) in surgical units, 41 (20.5%) in critical care units, and 55 (26%) in emergency departments (ED). For employment status, 53 (26.5%) were permanently employed and the others were working with contract. According to the nurses’ self-evaluation, the highest and lowest scores were belonged to the domains of ‘taking the health history’ and assessment of urogenital system respectively. Nurses received 84.87% and 16.37% of the scores in these two fields respectively (Table 1). 

Also, nurses’ believed that they have the highest proficiency in the domain of ‘taking the health history’ so that they got 87.25% of the score in this domain, while they scored themselves less proficient in the assessment of neurological system in which they got 34.58% of the score (Table 2). 

**Table 1.** Frequency of Using Different Health Assessment Skills

Frequency of Using Skills Based on Systems	The Min. and Max. Possible Score	Mean ± SD	Percent of Score
**History taking **	0 - 24	20.37 ± 4.61	84.87 ± 19.21
**Skin assessment**	0 - 16	10.55 ± 4.79	65.93 ± 29.99
**Head and neck**	0 - 56	15.65 ± 13.82	30.10 ± 26.57
**Ear, nose and eyes **	0 - 108	21.60 ± 22.63	20.00 ± 20.96
**Respiratory system**	0 - 32	9.28 ± 9.62	29.02 ± 30.06
**Cardiovascular system**	0 - 68	27.18 ± 16.98	39.97 ± 24.97
**Gastrointestinal system**	0 - 48	13.11 ± 13.57	27.31 ± 28.27
**Musculoskeletal system**	0 - 40	10.02 ± 10.75	25.06 ± 26.88
**Neurological system**	0 - 48	9.48 ± 11.28	19.76 ± 23.51
**Urogenital system**	0 - 40	6.55 ± 9.77	16.37 ± 24.44
**The total score**	0 - 480	139.68 ± 91.20	29.10 ± 19.00

**Table 2.** The Level of Proficiency in Different Health Assessment Skills

Proficiency in Different Skills	The Min. and Max. of Possible Score	Mean ± SD	Percent of Score
**History taking **	0 - 18	15.70 ± 3.54	87.25 ± 19.68
**Skin assessment**	0 - 12	8.89 ± 3.31	74.08 ± 27.61
**Head and neck**	0 - 42	20.50 ± 12.43	48.80 ± 27.55
**Ear, nose and eyes **	0 - 81	27.03 ± 22.31	25.02 ± 20.66
**Respiratory system**	0 - 24	10.75 ± 7.81	44.81 ± 32.70
**Cardiovascular system**	0 - 51	26.58 ± 14.26	52.12 ± 27.97
**Gastrointestinal system**	0 - 36	16.04 ± 11.48	44.56 ± 31.91
**Musculoskeletal system**	0 - 30	11.78 ± 8.75	39.26 ± 29.16
**Neurological system**	0 - 36	12.45 ± 9.63	34.58 ± 26.75
**Urogenital system**	0 - 30	12.87 ± 10.63	42.91 ± 35.43
**The total score**	0 - 360	163.00 ± 84.79	45.23 ± 23.46

Table 3 shows that nurses with permanent employment have got higher scores than nurses who were working with contract, subcontract or mandatory services, both in using and in mastering the health assessment skills (P = 0.58 and 0.21 respectively). No significant correlations were observed between the nurses’ age and the score they received in using (r = 0.05, P = 0.48) and mastery in health assessment skills (r = 0.08, P = 0.24). Also, no significant correlation was found between the nurses working experience and the score they received in using and mastery in health assessment skills (r = 0.12%, P = 0.10% and r = 0.05, P = 0.14 respectively). 

Male nurses had reported more frequency of using skills and proficiency in health assessment skills than female ones. However no significant association was observed between gender and frequency of using or proficiency in health assessment skills (P = 0.25 and 0.65 respectively). 

Table 4 shows that the total scores of nurses in critical care units were higher than other wards in the frequency of using skills. Also, the total score of nurses’ self assessment of their proficiency in health assessment skills was higher in EDs than nurses in other units. However, such differences was only significant between the nurses in EDs and surgery units (P = 0.02). Also, nurses in ED reported higher mastery in assessment of the nervous and urogenital systems than nurses in other units. However, such differences were statistically significant between the nurses in EDs and medical and surgical units (P = 0.03 and 0.004 respectively). 

**Table 3.** Frequency of Using Skills and Level of Mastery in Skills Based on Nurses Employment Status.

	Permanent employment, Mean ± SD	Contract, Mean ± SD	Subcontract, Mean ± SD	Mandatory services, Mean ± SD	P Value
**Frequency of using skills**	153.21 ± 80.33	135.47 ± 106.54	135.12 ± 85.63	136.60 ± 82.30	0.58
**Proficiency in skills**	163.43 ± 72.05	155.12 ± 89.36	179.87 ± 86.29	149.20 ± 88.77	0.21

**Table 4.** Self Assessment of Using and Proficiency in Health Assessment Skills in Different Units

	Medical, Mean ± SD	Surgical, Mean ± SD	Critical Care, Mean ± SD	Emergency, Mean ± SD	P Value
**Frequency of using skills**	143.55 ± 99.13	119.36 ± 85.46	162.07 ± 113.19	137.30 ± 60.13	0.16
**Proficiency in skills**	153.64 ± 86.35	144.74 ± 73.62	162.37 ± 94.66	190.96 ± 80.15	0.03

## 5. Discussion

The present study showed that nurses use their health assessment skills less than the desired level. Also the lowest rate of using was reported in skills related to assessment of urogenital system. This finding is consistent with the results of Wern et al. (14). Although the total use of the assessment skills was low; however, ignorance of assessing the urogenital system may be attributed to some of socio-cultural factors. It seems that questioning the patients about urogenital area and sexual behaviors and examining the urogenital areas are difficult and embarrassing for both nurses and patients. On the other hand, the same factors are probably affected on the performance of nurse educators. Then it can be assumed that nurse educators underestimated the education and evaluation of the skills in this area. Consequently their students had not opportunities to train, exercise and implementation of these skills, then they ignored and have forgotten the assessment skills in this area. Other studies have also reported that factors such as the students’ gender, age, religious beliefs, race, and trait features are important variables affecting such examinations (15). It has also been reported that nurses lacking of effective communication skills may negatively affect patients behaviors and make them to resist against being examined (15).

Most of the participants of this study reported that they are not proficient in health assessment. The lowest level of proficiency was reported in the assessment of the nervous system. This finding is consistent with the results of Wilds et al. (16). More than 80% of the nurses in the present study stated that they are proficient in history taking. However, in a study by Madani et al. the students in Zanjan Medical University were poorly to moderately skilled in history taking skills and reviewing the patients’ bodily systems (17).

In the present study, nurses working in emergency departments and the ones with higher ages and more service years stated that are more proficient in health assessment skills and use these skills more than other ones. In a study conducted by Mertoja et al. nurses with more age and more professional experience were also more proficient in health assessment (8, 18), however, Bahreini et al. have reported conflicting results (12). Nevertheless, it seems that by increasing the working experience, the nurses self-confidence has been increased that has subsequently lead them to estimate their own proficiency in health assessment higher and use these skills more. The more proficiency and more using of the skills by ED nurse may also be attributed to the nature of their work. They have more confrontations with emergency conditions, then they have delegated more authority and independence and consequently they had to be more proficient in health assessment skills. 

The present study showed that the frequency of using health assessment skills were higher in nurses with permanent employment status than the ones who were working with contract. This finding is also can be attributed to the level of professional experience. In Iran’s health system newly employed nurses are generally working with contract. They are usually newly graduated; less experienced and have low self-reliance. Such condition also may be influenced by the nursing education system. Some previous studies have also reported that the Iran’s nursing curriculum and nursing programs could not provide enough opportunity for nursing students to improve their knowledge and skills (19), and nursing students believed that the skills learned during education, do not meet their professional needs in work setting (20). Salehi et al. have also reported that the content and proportion of theoretical and clinical courses in nursing are not appropriate in Iran’s nursing programs (21). Another study has also reported that there is a significant gap between theoretical courses taught in universities and the tasks the nurses have to do in the practice settings (11). It has also been argued that most of the nursing textbooks are written based on the medical model and this is why the nurses can not apply them easily in practice (9). One study in New Zealand has also reported that factors such as lack of support from nursing supervisors, doctors and the hospital managers, heavy workload, lack of time and ambiguity in job description are obstacles to perform health assessment by nurses (4). Same reports are also available from Iran and show that in the current health system of Iran, number of nurses is lower than the standards; they have heavy workloads, ambiguous job descriptions and many writing tasks that keep them separate from patients and confined them within nursing stations (22). This situation not only hindered the nurses to use their own health assessment skills but also would lead to gradual depreciation of these skills. 

In conclusion, nurses in this study reported that use their own health assessment skills lower than desired level and reported that have undesired level of proficiency in this regards. This condition may be rooted in the nursing educational system, the physician centered structure of the health care system; ambiguous nurses job description, weak performance of the supervisory systems, and the low quality of the continual education system. Then, establishing appropriate continual education programs and workshops on health assessment skills, strengthening the supervisory systems, clarification of the nurses job decryptions, and revisions in the nursing education curriculums and educational contents to be more close to the needs of the practice setting and restructuring the health care system so that nurses can do the real nursing care and not accomplishing the tasks of the other groups may help to improve the health assessment of the nursing clients and the quality of nursing care.

Finally the results of this study should be considered cautiously as we relied on the nurses self reports both in the frequency of using and in the mastery in health assessment skills. Then, further observational studies are suggested in larger samples, different settings, and practical evaluation of these skills. 

## References

[1] Hanley E, Higgins A (2005). Assessment of clinical practice in intensive care: a review of the literature.. Intensive Crit Care Nurs..

[2] St-Onge C, Martineau B, Harvey A, Bergeron L, Mamede S, Rikers R (2013). From see one do one, to see a good one do a better one: learning physical examination skills through peer observation.. Teach Learn Med..

[3] Watson R (2002). Clinical competence: starship enterprise or straitjacket?. Nurse Educ Today..

[4] Lesa R, Dixon A (2007). Physical assessment: implications for nurse educators and nursing practice.. Int Nurs Rev..

[5] Weber J, Kelley J (2003). Health assessment in nursing..

[6] Dadgaran l, Parvizy S, Pyrovi H (2012). Aglobal issue in nursing students clinical earning :the theory-practice gap.. Procedia-Social Behav Sci..

[7] Delaram M (2006). [Clinical Education from the Viewpoints of Nursing and Midwifery Students in Shahrekord University of Medical Sciences].. Iran J Med Educ..

[8] Meretoja R, Isoaho H, Leino-Kilpi H (2004). Nurse competence scale: development and psychometric testing.. J Adv Nurs..

[9] Secrest JA, Norwood BR, duMont PM (2005). Physical assessment skills: a descriptive study of what is taught and what is practiced.. J Prof Nurs..

[10] Birks M, Cant R, James A, Chung C, Davis J (2013). The use of physical assessment skills by registered nurses in Australia: issues for nursing education.. Collegian..

[11] Giddens JF (2007). A survey of physical assessment techniques performed by RNs: lessons for nursing education.. J Nurs Educ..

[12] Bahreini M, Moattari M, Kaveh MH, Ahmadi F (2010). [Self assessment of the clinical competence of nurses in a major education hospital of shiraz university of medical sciences].. J Jahrom Univ Med Sci..

[13] Adib-Hajbaghery M, Karbasi-Valashani K, Heidari-Haratmeh A (2012). Correlation of Clinical Skills Self-Assessment of Nursing Internship Trainees with Their Teachers’ Evaluation.. Nurs Midwifery Stud..

[14] Wearn AM, Bhoopatkar H, Mathew TK, Stewart L (2013). Exploration of the attitudes of nursing students to peer physical examination and physical examination of patients.. Nurse Educ Today..

[15] Ferguson WJ, Candib LM (2002). Culture, language, and the doctor-patient relationship.. Fam Med..

[16] Wildes T, Anderson R (2004). The adult screening physical examination: what physicians do.. WMJ..

[17] Madani H, Bahraminejad N, Amini K, Rahimi A, Fallah R (2008). [Senior Nursing Students' Skills in Patients' Health Assessment in Zanjan University of Medical Sciences].. Iran J Med Educ..

[18] Meretoja R, Leino-Kilpi H, Kaira AM (2004). Comparison of nurse competence in different hospital work environments.. J Nurs Manag..

[19] Chang BL, Lee JL, Pearson ML, Kahn KL, Elliott MN, Rubenstein LL (2002). Evaluating quality of nursing care: the gap between theory and practice.. J Nurs Adm..

[20] Adib Hajbaghery M, Salsali M (2005). A model for empowerment of nursing in Iran.. BMC Health Serv Res..

[21] Salehi SH, Tavakol Z, Hasanzahraie R, Bashardost N, Mahjur SR (2001). [The performance evaluation of BS nursing graduates based on their own perspectives and their head nurses in the hospital affiliated to Isfahan University of Medical Sciences in 2002].. Iran J Med Educ..

[22] Nikbakht Nasrabadi A, Parsa Yekta Z, Emami A (2004). [Phenomenological study of nursing experiences in Iran and Sweden].. Hayat..

